# Participatory practices and participant observation together in action research: novel approaches to building capacity for health equity?

**DOI:** 10.3389/fpubh.2026.1779809

**Published:** 2026-05-01

**Authors:** Diana Campanella Schow

**Affiliations:** Department of Community and Public Health, Idaho State University, Pocatello, ID, United States

**Keywords:** capacity building, health equity, methods, participant observation, participatory practices

## Abstract

Health equity and the capacity to build it may be better achieved when the approaches of participatory practice and participant observation are more intentionally implemented together in applied, action research. An exploration of the literature compared results from a title and abstract search for the phrases participatory and participant observation. When searched for together, the number of articles that was retrieved was very low, flagging an issue worth consideration. A comparison of the approaches’ historical development, theoretical foundations, and practical applications sheds light on their similarities and differences. Participatory practices lean on teaching community members how to conduct action research; plan and implement projects, and advocate for themselves. Participant observation leans on researchers and evaluators being taught about different communities through processes of immersion and deep reflection. Both have positive and negative connections to health equity, capacity building, empowerment, and engagement. Revisiting these approaches as separate strategies for learning provides an opportunity to adapt and reinvigorate their coordinated and robust use, which could lead to novel approaches to action research and more successful treatment of power-imbalances that thwart health equity. Such a revisitation should include consideration of action projects that conceptualize the approaches side by side, actual versus intended use of coordinated approaches, methods for more leveraging of health infrastructure and healthcare education; and potential new approaches to participant observation. Woven throughout this article are the author’s perspectives and examples from 25 years of work on community-based, participatory action, ethnographic, and qualitative research, implementation, and evaluation projects.

## 1 Introduction

Participatory practices and participant observation, when intentionally implemented together, may lead to novel approaches that strengthen applied action research focused on capacity building for health equity. A description of capacity building and action research can provide context for why this proposition has merit. Capacity building is a process designed to strengthen the ability of individuals, organizations, communities and systems to better achieve their goals (1, 2). It can improve trust, relationships, and social capital–which is the glue for collective action with the goal of producing change (3). It can also increase basic resources, knowledge, skills, and health policies (4). Action research intentionally focuses on participatory engagement at individual, organizational, community and systems levels to encourage participants to address their own health issues (5). Common action-based activities include high and consistent contact between participants, transparency, critical reflection, and shared leadership. These strategies align with Robele’s (6) proposition that engaged, interactive environments are essential to generating shared understandings of health issues, which in turn can foster action. Capacity building and action research have also been combined (7, 8) to successfully improve health equity, which is defined as an opportunity to achieve “full health potential” (9, p. 8) through supportive/enabling conditions (10).

Participatory practices and participant observation are purpose-driven methods that are used to cooperatively produce knowledge and analyze and act upon social and cultural phenomena, like health equity (11–14). They too, are predicated on a high degree of engagement with individuals, organizations, communities, and systems of interest. And while participatory practices have been consistently included in capacity building and action research projects (15–20), this seems to occur less with participant observation (21–23). Further, very little appears to be known about the frequency with, and form in which, participatory practices and participant observation are implemented alongside each other in any circumstance whatsoever.

**Figure 1:**
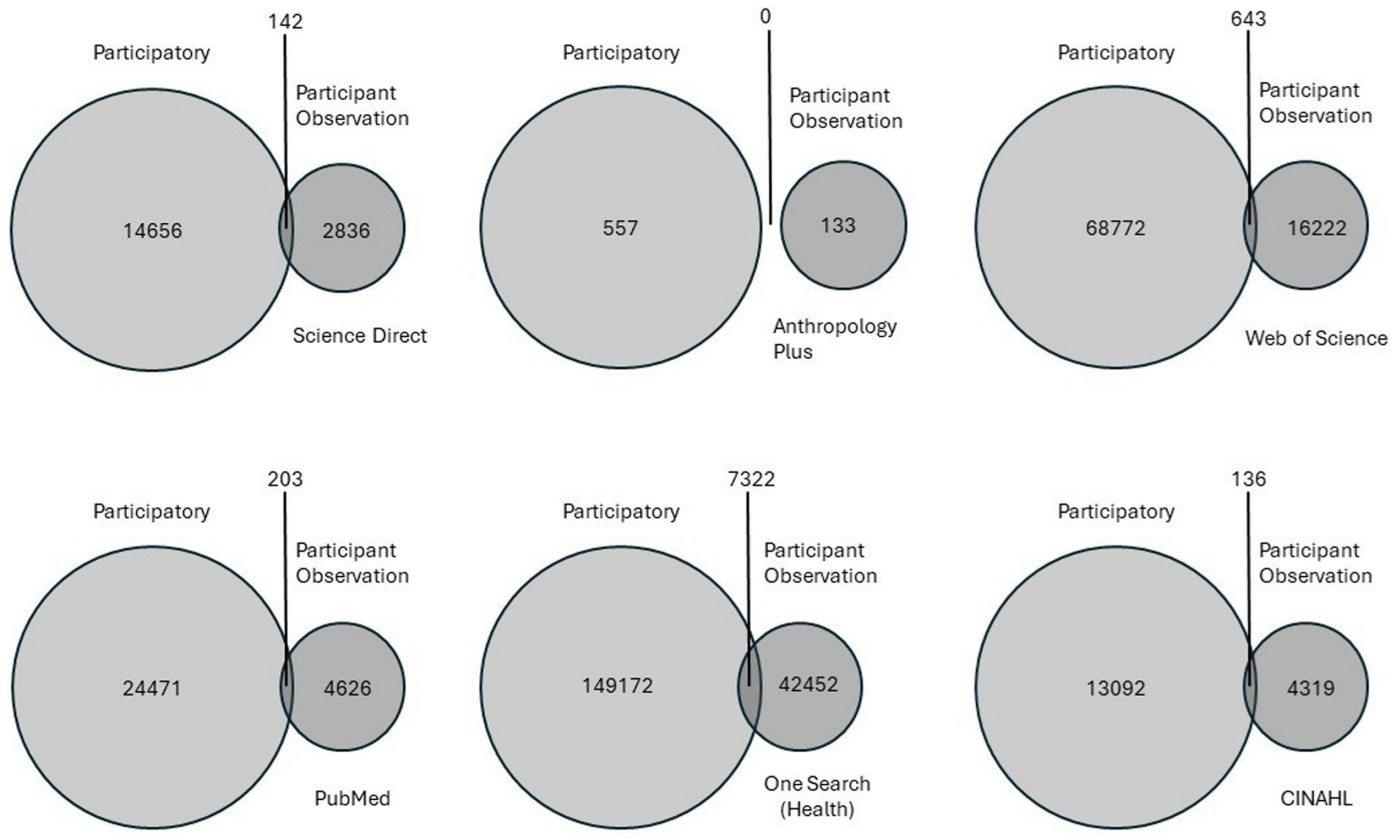
Results from exploration of six databases comparing the number of results in titles and abstracts that contained “participatory” or “participant observation” or “participatory” AND “participant observation.”.

In 2025, six databases were explored for the quantity of titles and abstracts that contained the phrases “participatory” or “participant observation,” or “participatory” AND “participant observation.” The databases were Anthropology Plus, CINAHL, One Search (Health), PubMed, Science Direct, and Web of Science. Results that included both phrases, with the word AND in between, were drastically lower than results that included just one of the phrases (Figure 1). Also, results that included “participatory” alone were much higher than results that included “participant observation” alone. This search was not exhaustive. Further systematic exploration of how these terms are used, in what combinations, and in what disciplines, may shed light on the expansiveness of their conceptualization. For example, the word “participatory” alone may be associated with a broader array of endeavors–which was the point of its choosing. This is worth exploring in systematic reviews to ascertain how participatory practices are framed within and beyond work done in the realm of health equity.

An example that does embrace the dual implementation of participatory approaches alongside participant observation is the work of Roque et al. (24), who implemented “community-based participant observation (CBPO).’ They published a step-by-step protocol for ensuring that participant observation aligns with the goals of community-based participatory research (CBPR), and they emphasized its relevance to the co-production of knowledge. Another example is the work of McGrath and Rudman (25), who used narrative interviews, semi-structured interviews, and strategic observation sessions to understand the experiences of older adults with low vision. Longer narrative interviews provided opportunity to more fully consider context. Observation sessions were chosen by participants and were time-bound, which avoided the need for researchers to “arbitrarily ‘hang out (3, p.4).’” These types of examples seem to be limited, however, which leaves little opportunity to analyze their feasibility and effectiveness; let alone to explore their potential to make a substantial difference in health equity and the power imbalances that foment it.

My personal experience of working with these approaches over 25 years with more than 14 health topics bears this out. I have seldom seen participatory approaches and participant observation side by side, which begs the question, “Why?” Following is a discussion of both as they relate to capacity building, engagement, and their somewhat distinct treatment of what it means to participate. They lean away from each other in some fundamental ways. Also addressed are successes and challenges associated with the approaches, recommendations for future directions, and potential implications of their collective use in capacity building and action research.

## 2 Participatory practices

Participatory practices are about teaching and engaging community members in activities that are familiar to researchers. In this way, community members participate in learning how to conduct, implement, and analyze research. They also engage in empowerment activities and collectively develop innovative solutions to complex social problems (26), which are critical to development of the self in relation to society (27).

The concept of participatory practice is derived from traditions that focus on change. Wallerstein and Duran (26) provide an account of the historical and theoretical development of change-focused, action-oriented traditions like participatory action research (PAR) and community-based participatory research (CBPR), where participatory practices are prioritized. For this discussion, PAR, CBPR, and participatory practices should not be conflated, but it is important to forefront their very close and interdependent relationship. Participatory practices have roots in the sociological, organizational, and pedagogical work of Kurt Lewin and Paulo Freire who called for engagement of community members and workers to impact change by developing sustainable trust, treating them as equal partners, and fostering environments that support shared learning (28, 29).

Lewin’s approach to organizational change involves accounting for resistance to change (freezing), creating a safe environment in which to explore new ideas and change (unfreezing), and supporting sustainability of change (refreezing) (30). Masterton et al., (30) concluded that this approach has merit in the introduction and adoption of the controversial, yet evidence-based practice of harm reduction. Hassan Ahmed et al. (31) recently used Lewin’s Change Management Theory to successfully “unfreeze, change, and refreeze (p. 335)” nurses’ approaches to discharge planning. The project supported a participatory educational process that built trust between nurses and patients, inspired stronger engagement from nurses in discharge planning, and set a tone for integrating and sustaining change through positive reinforcement of patients.

Freire’s approach to education eschews the didactic, lecture-based learning environment as a means to achieve social change. Instead, it promotes interactive learning, which allows for shared, critical examination of social complexities that drive health inequities – from structural inadequacies to racism (32). Ultimately this promotes a raising of consciousness to problems and solutions for communities and for teachers and researchers. Heideman et al. (33), for example, recently used Freire’s pedagogical approach to engage a multidisciplinary group of healthcare providers to raise consciousness about challenges and potentialities of working in primary care settings, thus empowering them to act on important issues.

When implemented through CBPR, participatory practices have been shown to support capacity building in the areas of economics, technology, skills, socialization, and politics (34). In the realm of health and CBPR, they have been lauded for their ability to empower, promote health equity, raise consciousness among researchers and participants, and ultimately support change through collective learning and action. They can also help improve health outcomes (35–38) and the situations within which those outcomes are made manifest (36, 39).

Even so, there are challenges when it comes to effectively implementing participatory practices. They are time consuming, and sustained engagement is difficult to attain. Although Yau et al. (35) found an association between CBPR and positive mental-health outcomes, they also concluded that adherence to CBPR participatory practices is lacking, which can thwart the ability to build capacity. Of the 14 studies included in their systematic review 71% had “low adherence to community involvement in the research process (p.13)”. Eight of the studies were rated as low contributors to capacity building, and none were rated as high.

My own work in action research corroborates these challenges. Between 2002 and 2006, the Hispanic Health Projects in southeastern Idaho was led by a director who maintained strict allegiance to core CBPR principles (40). The program successfully trained community health workers *(promotoras de salud)* to do research and engage in promoting health. It provided opportunities to advance their education and to make healthcare more accessible. It raised awareness about type 2 diabetes prevention, body image, healthy cooking, physical activity, and women’s health. It was the most adherent work I have ever done as it relates to CBPR, and it embraced the integration and mutual respect that drives motivated participation. Even so, some activities were limited by funding restrictions, reporting guidelines, and short timelines that constantly challenged our ability to comprehensively support participation. This was the case with other projects I have worked on as well (41–43). Our intentions did not always lead to implementation.

Along these lines, in a recent scoping review, led by Frahsa et al. (44), our team explored community participation in public health research during the COVID-19 pandemic. To improve participatory practices, we offered eight recommendations including being transparent about levels of participation when writing results and reporting on the amount and type of participation during different phases of a project. Even if implemented effectively, however, participatory practices alone cannot do all things.

## 3 Participant observation

This is where more deliberately integrating participant observation into participatory research has promise. Participant observation is about teaching researchers and engaging them in activities that are familiar to community members. In this way, researchers participate by, for example, studying historical contexts, writing field notes, observing rituals and rites of passage; examining artifacts, listening to stories, and embodying what occurs through apprenticeship opportunities.

Participant observation has roots in anthropological research (i.e., ethnographic field work) (45, 46), and it takes place over long periods of time. It requires immersion in communities of study and is theorized to result in better understandings of cultures and phenomenological experiences. For example, Cushing was an ethnographer who lived with the Zuni people in New Mexico for 5 years between 1879 and 1884 (47). He became skilled at making Zuni artifacts and observing and participating in their belief systems. His works documented what he referred to as Zuni folk tales, fetishes, and creation myths (48). He immersed himself so fully in the culture that he was adopted as a Zuni warrior.

However, it is no secret that early anthropological projects like Cushing’s were built on racist, and colonial perspectives about “other” cultures. These issues are addressed at length in other publications (49, 50), and they should never be forgotten; especially considering current political tensions that seem to be exacerbating racism (51).

It is fortunate that participant observation has been used in recent decades to call out and address inequities (52) and to challenge patronizing and racist actions (53). In 2021, the American Anthropological Association issued a formal apology to Indigenous communities for decades of disrespect on behalf of the profession (54). In 2024, Herkshan et al. (54) called for true, rather than in-name-only, Tribally-responsive research engagement and more public acknowledgement of past research-based harms. Also in 2024, Horrill et al. (55) called for a revisitation of participant observation in health equity research to ensure it is approached through “trauma-, violence-, and justice-informed (p. 1)” lenses. In that same year, Seim built on the works of others to call for a more reflexive “observant participation (56, p.121)” to ensure, among other things, that what is observed when a researcher is immersed in another’s social context is represented only after deep reflection about the researcher’s assumptions and biases.

Through completion of a doctoral dissertation between 2011 and 2017, I had the time to engage in this more thoughtful type of participant observation to address my research topic - capacity building and promotion of physical activity among vulnerable groups in the European Union. The project supported visits to multiple countries and immersion in the contextual and social situations where physical activity was being promoted-even if only for a short while.

The process forefronted the social knowledge and expertise held by the individuals and organizations I collaborated with. I was invited to museums and cultural events. I learned about egg painting and the lingering effects of the Ceaușescu regime while driving through the Romanian countryside. I walked on a gravel soccer pitch in a Romanian prison and saw inmates fall and skin their knees. I participated in game nights with gang members in Scotland, where in contrast to Romanian prisoners, they played on lush, artificial soccer pitches. I also learned about the importance of Irn-Bru to Scottish culture. I got caught up in Vogue night in Portugal, and observed housing insecure individuals play soccer on less-than-new tennis courts; and after observing aging citizens playing on the monkey bars in Latvia, I was escorted to the theater where Baryshnikov trained. I believe these tactile and sensory episodes increased mutual trust and helped me learn with humility, connecting more fully with the lived experiences of my partners. They provided a window into strengths and injustices that I would not have awoken to in more formal settings, which supports Freire’s point, “solidarity requires that one enter into the situation of those with whom one is solidary (29, p.49).” Such levels of engagement could be extremely valuable to building health equity and capacity.

Much like ethnography, dissertation field work may allow deeper exploration of social and contextual complexities because it not only insists on immersed interaction with the subject matter, it also frees the writing process itself. Page numbers are generally not a concern. The goal is to write more–more broadly and more deeply. When writing at length, we can thickly describe (57) situations so they can be more fully understood before re-presenting them to policy and decision-makers.

## 4 Combining the approaches for capacity building

In addition to participant observation strategies, my dissertation project team also adhered to many participatory practices like shared decision-making, co-designing research questions, building interventions, analyzing outcomes, and disseminating meaningful results (58). In this sense, we co-developed best practices, convened international meetings, conducted interviews, facilitated focus groups, and engaged in group planning sessions. We went to conferences together and took group photos. These activities adhered strictly to the subject of capacity building and supported the improvement of programmatic approaches to addressing chronic disease through physical activity among 13 participating countries and organizations.

This was different than what I explored and learned about through participant observation; that being the broader social and historical milieu within which capacity building and physical activity promotion were being implemented. In my opinion, this was important because capacity, or lack thereof, can “only be understood by learning how social actors perceive and respond to particular needs and demands at individual, community, organizational, or institutional levels (59 p. 29).” Through the combination of approaches, time was spent researching all of these levels. This forced a confrontation with the reality that capacity itself is socially constructed and situational. It also served as a stronger form of triangulation for the project. It provided more and different opportunities to build trust, support collective learning, and understand partners in a much broader and more profound sense.

Following these insights and reflections, Table 1 is presented as an initial attempt to conceptualize the practical leanings of participatory practices alongside those of participant observation. There are no lines between columns or rows. This represents the fluidity of the leanings within and between categories. It is acknowledged that there are many approaches to, and interpretations of, these leanings. Their various forms align with multiple theories and disciplines, which should be highlighted in future work. The list is not exhaustive. It is respectfully presented as a first step in acknowledging the collective history and potential of its contents.

**Table 1:** A three-column comparison of the theoretical and practical leanings of participatory practices on the left, participant observation on the right, and similarities between both in the middle.

Action research		
Participatory practice (PP) leanings	PP & PO leanings - actuals and potentials	Participant observation (PO) leanings
	Trust & rapport	
	Reflexivity	
	Active listening	
	Participation and engagement	
	Cultural humility	
	Accurate representation of voice	
Public health and population health	Social sciences	Humanities
Community members learn about research and evaluation methods and self-advocacy		Researchers learn about skills, rituals, rites of passage, values, beliefs and lifeways
Shared decision-making		Deep historical archaeology
Group projects and activities		Cultural immersion and living, working, and eating together - tactile engagement
Partnership development	Leverage health care infrastructure – connect evaluation and research	Long-term observation and strategic observation – creating intimate connections
Gather Data (e.g., semi-structured interviews, focus groups, interactive workshops, biodata, dietary recalls, surveys, photos, audio, asset maps)	Observant participation Photos, audio Participants observe researchers	Gather data (e.g., ethnographic field notes, observations, conversations, oral histories, unstructured interviews, photos, audio, case studies)
Empowerment		Apprentice
Create forums and places at the table for community members to share perspectives and make decisions		Shadow (i.e., closely observe for an extended duration) to watch how community members implement perspectives and make decisions
Create asset maps	Participatory transects Environmental scans	Examine artifacts
Research products – white papers, policy briefs, journal articles, poster presentations, conference papers, implementation plans, community declarations, Photovoice conclusions	Digital Storytelling	Research products – ethnographies, historical non-fiction, documentaries journal articles, conference papers, poster presentations, traveling photo displays

## 5 Discussion

Moving forward, the impacts of coordinating the practices could be far reaching in terms of creating more, safer and novel opportunities for dialog, more robust and novel forms of triangulation, and better health equity outcomes. As discussed above, we should be cognizant of our capacity to do so as we assess actual versus intended use of coordinated approaches. This is where leveraging health care infrastructure and education may be helpful in developing new approaches to participant observation. Also, at the core of any exploration should be a discussion about ethics, how issues of power may reveal themselves, and how consent from all participants is explicitly ensured.

Leveraging the many health care and educational programs that exist may increase shared capacity. In Idaho, for example federally funded health clinics, hospitals, non-profits and public health districts promote a multitude of health equity programs. Idaho also maintains a robust cadre of health professions education programs. Partnering to integrate more fully into their evaluation infrastructure might influence a stronger, more long-term action research foundation that includes participant observation.

Regarding new approaches to participant observation, there is opportunity to balance more power in action research. Action researchers often rely on community representatives, or bridge people (60), to help gain trust and develop credibility with communities. They also implement participatory practices to train community representatives to conduct projects. These same practices could be used to train community members to use participant observation to observe researchers, partners that have power (e.g., doctors and educators), and other community members. If trust is the goal, researchers might allow themselves to be just as scrutinized as the individuals and communities they are working with. Rotating the observers’ gaze all the way around, 360 degrees, has the potential to foster more social participation, build more trust, and empower communities in newer ways that really do build capacity. If done incorrectly, however, it could be damaging. Much consideration should go into development of this idea.

In the future, we can do better when it comes to building capacity for health equity through action research. We can leverage existing funding systems and programs, model projects on longer time frames, expand observation practices to include researchers and others as subjects; more openly acknowledge where, despite best efforts, we have fallen short when it comes to true participatory engagement; and more intentionally combine participatory practices with participant observation.

## Data Availability

The raw data supporting the conclusions of this article will be made available by the authors, without undue reservation.
